# The Roles of Featural and Configural Face Processing in Snap Judgments of Sexual Orientation

**DOI:** 10.1371/journal.pone.0036671

**Published:** 2012-05-16

**Authors:** Joshua A. Tabak, Vivian Zayas

**Affiliations:** 1 Department of Psychology, University of Washington, Seattle, Washington, United States of America; 2 Department of Psychology, Cornell University, Ithaca, New York, United States of America; University of Minnesota, United States of America

## Abstract

Research has shown that people are able to judge sexual orientation from faces with above-chance accuracy, but little is known about how these judgments are formed. Here, we investigated the importance of well-established face processing mechanisms in such judgments: featural processing (e.g., an eye) and configural processing (e.g., spatial distance between eyes). Participants judged sexual orientation from faces presented for 50 milliseconds either upright, which recruits both configural and featural processing, or upside-down, when configural processing is strongly impaired and featural processing remains relatively intact. Although participants judged women’s and men’s sexual orientation with above-chance accuracy for upright faces and for upside-down faces, accuracy for upside-down faces was significantly reduced. The reduced judgment accuracy for upside-down faces indicates that configural face processing significantly contributes to accurate snap judgments of sexual orientation.

## Introduction

People are able to judge men’s and women’s sexual orientation with above-chance accuracy relying on no more than grossly impoverished facial photographs (i.e., grayscale, hair-removed) presented for as few as 40–50 ms [1], [2], [3], [4], [5]. Despite the growing literature on reflexive, intuitive, rapid, or “snap” judgments of sexual orientation from faces, little is known about the processes by which these judgments are formed. When making accurate judgments of sexual orientation, do people rely solely on the processing of individual facial features (i.e., featural face processing)? Do people rely on the processing of relationships among facial features (i.e., configural face processing)? Or, do people rely on some combination of both? Investigating the face processing mechanisms governing sexual orientation judgments has implications for understanding whether sexual orientation is judged as category-based (e.g., male vs. female; black vs. white) or identity-based (e.g., familiar vs. unfamiliar) person information. Testing the roles of configural and featural face processing on the accuracy of snap judgments of sexual orientation – that is, rapid and intuitive judgments of sexual orientation – is the primary goal of the present research.

Existing work investigating *what* type of social information underlies judgments of sexual orientation from faces has indicated that gender atypicality, whether natural or manipulated via morphing software, makes faces more likely to be perceived as gay or lesbian [6], [7]. Other research focusing on *where* in the face valid cues reside has shown that the mouth and eye areas, alone, enable above-chance accuracy in sexual orientation judgments [2], [3]. Despite this growing body of research, work has yet to examine *how* the face is processed to give rise to reliable judgments of sexual orientation. Research indicates that there are two routes for perceiving the human face: *featural processing* primarily encodes individual facial features (e.g., an eye or nose), and *configural processing* primarily encodes relationships among featural cues (e.g., distance between the eyes) [8], [9], [10], [11], [12], [13].

It is important to clarify the distinction between the *how* (face processing) question and the *where* (in the face) question, which has been addressed in previous research [2], [3]. For example, Rule et al. [2] showed that men’s sexual orientation could be judged with above-chance accuracy from the eye area alone or the mouth area alone, and that accuracy for either of these areas of the face was lower than accuracy for the whole face. At first glance, these results may suggest not only that judgments of sexual orientation involve certain facial areas, but also that configural face processing (whole faces) raised accuracy above the accuracy enabled by either of the individual areas of the face. However, an alternative explanation is that the individual facial areas provide at least partially independent sources of sexual orientation information and that when presented simultaneously (as a whole face), judgment accuracy increased (compared to accuracy for each area alone) simply because more featural information was available (but not necessarily because any configural processing occurred). Thus, to date, the role of configural face processing in judgments of sexual orientation judgments is unknown.

Configural processing can refer to several distinct ideas. Following the definition provided by Maurer et al. [9], in this paper, we refer to configural processing as any or all of the following: (a) processing the ordinal spatial relationships among individual features (e.g., eyes appear above noses), (b) processing the cardinal spatial relationships among individual features (e.g., the amount of space between the eyes), or (c) processing the face in a holistic or gestalt manner (i.e., the general shape of the face). All three subtypes of configural face processing are diminished by facial inversion; disentangling these subtypes of configural face processing is beyond the scope of this paper. Of note, specific areas of the face (e.g., pairs of eyes) can possess both featural cues (e.g., an eye) and configural cues (e.g., distance between eyes) (for a review of different types of face processing, see [9]). Thus, strictly speaking, previous work showing that individual areas of the face can enable above-chance sexual orientation judgments [2], [3] does not indicate whether judgments of each face area were driven by featural processing, configural processing, or both.

Comparing the accuracy of judgments made from facial photographs presented upright vs. upside-down is one method for determining whether configural face processing contributes to a character judgment [14]. Displaying photographs of faces upright allows for unimpeded processing of both featural and configural facial cues; in contrast, displaying facial photographs upside-down severely disrupts processing of configural facial cues but has little [14], [15] or no detectable effect on featural face processing [8], [9], [10], [11], [12], [13], [16], [17], [18], [19], [20], [21], [22], [23], [24]. More concretely, when faces are manipulated so that they differ in featural information (e.g., shape of eyes, nose or mouth [8], [21]; eye color [22]; combinations of eye color and hair color [8]; brightness of individual facial features [8]), individuals are able to distinguish faces (i.e., by making same vs. different judgments when first seeing or when recalling faces) when they are presented upright as well as upside-down [8], [21], [22], [23]. However, when faces are manipulated so that they differ in configural information (e.g., distance between nose and mouth [8]; mouth or eye position [21], [22]; interocular distance [23]), individuals are only able to distinguish faces when faces are presented upright [8], [21], [22], [23]; see [9], p. 257, Figure 3.

Research and theorizing by Cloutier and colleagues suggests that understanding face processing mechanisms (i.e., featural and configural) has implication for various social inferences, with social categorization (e.g., male vs. female; black vs. white) relying heavily (but not necessarily exclusively, e.g., [13]) on featural processing, and identity judgments (e.g., familiar vs. not; famous vs. not) relying heavily (but not necessarily exclusively) on configural processing. Using this facial inversion technique, Cloutier, Mason, and Macrae [12] showed that judgments of sex could be accurately rendered when faces were both upright and upside down. In contrast, judgments of fame (famous vs. not famous) could be accurately rendered when perceivers viewed target faces upright but were significantly less accurate when the faces were presented upside-down–i.e., when configural face processing is dramatically impaired, e.g., [8], [13], [19], [21], [22], [23], [24]. Because “the extraction of featural information is largely resistant to the effects of inversion” [12] (p. 886), the researchers concluded that judging fame requires configural face processing – which shows reliably large effects of facial inversion.

### The Present Research

Does configural face processing contribute to accuracy of sexual orientation judgments? Understanding the processing that allows sexual orientation to be read from faces may reveal how sexual orientation, as a social construct, is conceptualized. Whereas featural face processing is sufficient to enable judgments of social category information (e.g., male vs. female; black vs. white), configural face processing is necessary to enable judgments of social identity information (e.g., familiar vs. unfamiliar; famous vs. not famous) [12], [14], [16]; see also [18]. In contrast to categories such as race or gender, sexual orientation is less obvious. Thus, it is unclear whether individuals would rely on category (featural) face information, individuating (configural) face information, or both.

Additionally, we investigated the effects of stimulus gender in the ability of participants to make reliable sexual orientation judgments. Previously, snap judgments of sexual orientation have been examined separately for men’s and women’s faces. Casual comparison of accuracy rates across papers, i.e., [1], [2] vs. [3], implies that women’s sexual orientation may be judged more accurately than men’s, but direct comparisons have not been performed to date. The possibility that judgments of sexual orientation differ as a function of gender is likely given the well-established gender differences in experiences of romantic love and sexual desire, neurophysiological and hormonal responses to sex and attachment, and phenomenology of sexual orientation, e.g., [25], [26], [27], [28], [29], [30], [31]. Thus, we predict that facial markers of sexual orientation may differ by gender, as well.

### Hypotheses

#### Hypothesis 1

Configural face processing contributes to accurate snap judgments of sexual orientation. Because sexual orientation is phenotypically ambiguous, we predicted that the deeper, more individuating type of face processing – configural face processing – would contribute to judgment accuracy. In practical terms, this means that judgment accuracy should be reduced when faces are presented upside-down (vs. upright).

#### Hypothesis 2

The process of reading sexual orientation from faces may differ as a function of whether the stimulus person (face) is male or female. In the present experiments, participants judged both men’s and women’s faces, allowing for direct comparisons of judgments as a function of target gender. This hypothesis is exploratory in nature and does not carry a directional prediction.

## Experiment 1

Can sexual orientation be read from briefly presented faces of men and women? And, does accuracy differ for reading sexual orientation from men’s vs. women’s faces?

### Method

#### Ethics statement

This research protocol was reviewed and approved by the Institutional Review Boards for Research with Human Participants at the University of Washington and at Cornell University. Participants provided written informed consent prior to engaging in research activities. This research was conducted in accordance with the standards set forth by the American Psychological Association.

#### Participants

Twenty-four University of Washington students (19 women; age range = 18–22 years) participated in exchange for extra course credit. Data from seven additional participants were excluded from analyses due to failure to follow instructions (*n* = 4) or computer malfunction (*n* = 3).

#### Apparatus

Inquisit 3.0.3.2 [32] was implemented on Windows XP-based computers with 17-inch CRT monitors (1024×768 resolution and 60 Hz refresh rate).

#### Facial photograph selection and preparation

The stimulus set included facial photographs of 111 gay men, 122 straight men, 87 gay women, and 93 straight women. Facial photographs were gathered from Facebook.com profiles, cf. [1], [3] of individuals living in 11 major US cities who self-identified as straight or gay; photographs of self-identified bisexual people were not used as target stimuli. Each photograph had been posted by a target or a target’s friend.

To collect and standardize the photographs used in the present research, we trained 11 undergraduate research assistants who were kept blind to the experimental topic and hypotheses to perform all photograph collection and preparation. At the conclusion of the stimulus preparation process, each research assistant was probed for guesses about the research purpose; no guess was accurate. Because research assistants who were blind to the experimental topic and hypotheses collected the stimuli, we decreased the possibility of introducing unintentional experimenter effects in our stimulus set [33], [34]; see also [35].

More specifically, we provided research assistants with detailed instructions about which photographs could be included in the stimulus set (e.g., exclude photos of minors, exclude photos with facial jewelry such as eyewear; full instructions available from the first author upon request). Research assistants were instructed to follow the instructions carefully and to select photos for the stimulus set only according to the instructions. To minimize the prospect that non-face cues would influence judgments, photographs of men or women with facial alterations or adornments (e.g., scars, eyewear, facial hair, makeup, non-earlobe piercings, etc.) were not included as experimental targets. To maximize consistency across faces, only photographs of White-appearing individuals who self-identified ages of 18–29 were included.

**Figure 1:**
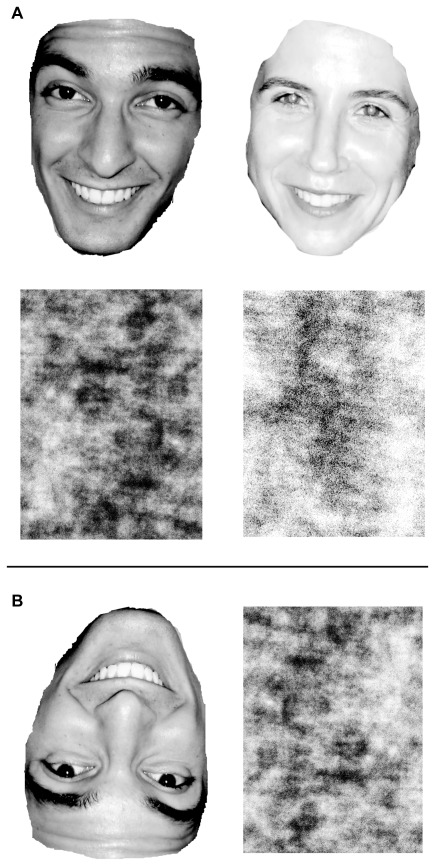
Sample stimuli from the sexual orientation detection task. (a) Example female face and backward mask (Experiments 1 and 2) and example male face and backward mask (Experiment 1); (b) lightened male face and backward mask (upside-down; Experiment 2).

Using Adobe Photoshop CS3 Extended, research assistants removed hair and ears from each head and converted each image to grayscale (8-bit bitmap format), leaving the final “face” stimulus (Figure 1a). When presenting faces to participants, Inquisit standardized each image’s height to 200 pixels and adjusted each photograph’s width proportionally, resulting in undistorted images of nearly constant size. For illustrative purposes, it is worth noting that given our apparatus (17-inch 4×3 aspect ratio CRT monitors with 1024×768 pixel resolution), 200 pixels (the height of each face image stimulus) is approximately equal to 26% of the total vertical screen space, or about 2.90 inches.

#### Sexual orientation judgment task

Each trial consisted of: (a) a fixation cross for 1000 ms, (b) a target face stimulus for 50 ms, and (c) a backward mask for 100 ms, after which participants categorized the target face as either “gay” or “straight” “*as quickly and accurately as possible”* by depressing “A” or “L.” The intertrial interval was 1000 ms.

Faces of women were, on average, lighter than faces of men. Therefore, we created four masks to match the luminance of female targets, and four masks to match the luminance of male targets. Masks were generated using Matlab R2008b by adding random noise to the white areas of a facial photograph and then randomizing all of the pixels in the photograph such that spatial frequency gradients were held constant (with respect to the original face image). The resulting masks appear to be randomly assorted pixels, but, in fact, contain light-dark gradients that are equiprobable to those found in the original faces (Matlab m-code modified from [36]; Matlab m-code available from the first author upon request; see Figure 1).

Judgments for male and female targets were made in separate 112-trial blocks. Each block consisted of 96 randomly-ordered faces (48 gay and 48 straight; randomly selected without replacement from all faces of each type) and 16 control trials. To monitor participants’ attention, we included control trials that unambiguously represented either the category gay or straight (i.e., an image of two same-gender or opposite-gender stick figures holding hands). Block order (female faces first vs. second) and response keys (“gay” on left vs. right) were counterbalanced across participants; these procedural factors did not produce any significant main effects or interactions and are not discussed further.

#### Data analytic strategy

We measured sexual orientation judgment accuracy using A′ [37], a nonparametric measure of signal sensitivity. A′ measures sensitivity to the signal “gay” after correcting for participants’ biases to categorize faces as straight or gay. A′ is interpreted on a probability scale, with chance responding indexed by an A′ of .5; accordingly, A′ may be interpreted as a bias-adjusted accuracy score. Two A′ scores were computed for each participant: A′_f_ (women’s faces) and A′_m_ (men’s faces). To confirm our findings, all analyses were repeated using d′ (a parametric index of signal detection) as the dependent measure; the results were unchanged. In signal detection analyses (e.g., the computation of A′ or d′), there are two components of accuracy: the hit rate (reported in this study as H_f_ and H_m_), or the proportion of gay faces correctly perceived as gay, and the false alarm rate (reported in this study as FA_f_ and FA_m_), or the proportion of straight faces incorrectly perceived as gay.

A preliminary mixed-model analysis of variance (ANOVA) on accuracy with target gender as a repeated-measures factor also included participant sex as a between-participants factor; participant sex did not produce any significant main effects or interactions, consistent with previous work, e.g., [1], [3], and was dropped from analyses. One-sample *t*-tests examined whether accuracy of judging sexual orientation from men’s and women’s faces was better than chance. A paired-samples *t*-test examined differences in sexual orientation detection accuracy as a function of target gender. All *p*-values reported in this paper are based on two-tailed tests.

### Results and Discussion

**Figure 2:**
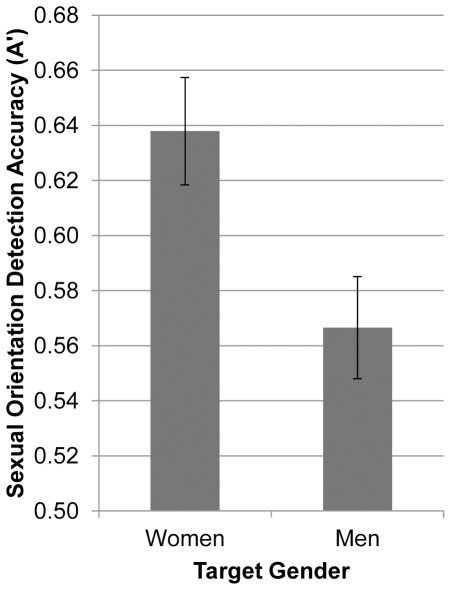
Accuracy of detecting sexual orientation from upright faces (Experiment 1). Mean accuracy (A′) in judging sexual orientation from faces presented for 50 milliseconds as a function of the target’s gender (Experiment 1). Error bars represent ±1 *SEM*.

As displayed in Figure 2, participants were significantly better than chance at reading women’s sexual orientation (*Mean* A′_f_ = .64), *t*(23) = 7.07, *p*<.001, Cohen’s [38] effect size *d* = 1.44. Participants also read men’s sexual orientation significantly better than chance (*Mean* A′_m_ = .57), *t*(23) = 3.58, *p*<.002, *d* = 0.73. This finding indicates that naïve perceivers can, in fact, read sexual orientation from unknown others’ faces.

Additionally, faces of women were judged more accurately than faces of men, *t*(23) = 2.74, *p* = .01, *d* = 0.55 (Figure 2). There was no significant difference in the hit rate for women’s faces (*Mean* H_f_ = .38, *SD* = 0.17) and men’s faces (*Mean* H_m_ = .42, *SD* = 0.12), *t*(23) = −1.38, *p* = .18, *d* = −0.28. However, the false alarm rate was significantly lower for women’s faces (*Mean* FA_f_ = .25, *SD* = 0.16) than men’s faces (*Mean* FA_m_ = .36, *SD* = 0.14), *t*(23) −3.83, *p*<.001, *d* = −0.78. This target gender difference is intriguing and contrary to cultural expectations; given the relative prominence of representations of the concept “gay man” vs. the concept “lesbian” (e.g., in the media; [39]), we might have expected a target gender effect in the opposite direction. Before theoretically interpreting this finding, it is worth noting that faces of men were darker than the faces of women. This difference in luminance for men’s and women’s faces reflects an actual (population) gender difference in face brightness. That is, gender differences in facial hair, even among clean-shaven individuals, lead to gender differences in luminance of facial photographs [40]. We elected to conduct Experiment 1 without equating men’s and women’s faces on luminance in an effort to preserve the original images. Nonetheless, the target gender difference in luminance may be one reason why the accuracy of female targets was higher than that of male targets. To rule-out this possibility, we equated the luminance of men’s and women’s faces in Experiment 2 and retested Hypothesis 2. We also introduced an experimental manipulation of face spatial orientation (upright vs. upside-down) to test the roles of configural and featural face processing in snap judgments of sexual orientation (Hypothesis 1).

## Experiment 2

To what degree does the ability to read sexual orientation from women’s and men’s faces depend on configural face processing? To answer this question, we capitalized on the facial inversion effect [19]. To the extent that perception of sexual orientation from faces relies on configural cues, accuracy of sexual orientation detection should deteriorate when faces are presented upside-down (vs. upright). We also investigated whether sexual orientation was read more accurately from faces of women (vs. men) when luminance was equated across genders.

### Method

#### Ethics statement

This research protocol was reviewed and approved by the Institutional Review Boards for Research with Human Participants at the University of Washington and at Cornell University. Participants provided written informed consent prior to engaging in research activities. This research was conducted in accordance with the standards set forth by the American Psychological Association.

#### Participants

One hundred twenty-nine University of Washington students (92 women; age range = 18–25 years) participated in exchange for extra course credit. Data from 16 additional participants were excluded from analyses due to failure to follow instructions (*n* = 12) or average reaction times more than 3 *SD* above the mean (*n* = 4).

#### Apparatus

Same as Experiment 1.

#### Facial photograph preparation

The set of faces used in Experiment 2 was the same as that used in Experiment 1. Faces of women were, on average, lighter than faces of men. In order to equate luminance of men’s vs. women’s photographs, we increased the luminance of each pixel in images of men’s faces by 11% using Matlab R2008b (Matlab m-code available from the first author upon request; see Figure 1b). Masks were generated using the same process described in Experiment 1.

#### Sexual orientation judgment task

As in Experiment 1, each trial consisted of: (a) a fixation cross for 1000 ms, (b) a target face stimulus for 50 ms, and (c) a backward mask for 100 ms, after which participants categorized the target face as either “gay” or “straight” “*as quickly and accurately as possible*” by depressing “A” or “L.” The intertrial interval was 1000 ms.

Judgments for men’s and women’s faces were made in separate 112-trial blocks. Participants were randomly assigned to judge upright faces (*n* = 67) or upside-down faces (*n* = 62) (i.e., photographs and masks that had been rotated 180°; see Figure 1b). (Spatial orientation (upright vs. upside-down) was initially a repeated-measures variable; however, due to order effects (*p*<.05), it was only possible to interpret data from the first of the two conditions. Accordingly, we report spatial orientation as a between-participants variable and only present data from the condition each participant completed first.) Each block consisted of 96 randomly-ordered faces (48 gay and 48 straight; randomly selected without replacement from all faces of each type) and 16 control trials. To monitor participants’ attention, we included control trials that unambiguously represented either the category gay or straight (i.e., the word “gay” or “straight”). Block order (women’s faces first vs. second) and response key assignment (“gay” on left vs. right) were counterbalanced across participants; these procedural factors did not produce significant main effects or interactions and are not discussed further.

#### Data analytic strategy

For each participant, two A′ scores were computed: A′_up_w_ (upright women’s faces) and A′_up_m_ (upright men’s faces), *or* A′_ud_w_ (upside-down women’s faces) and A′_ud_m_ (upside-down men’s faces). One-sample *t*-tests examined whether accuracy of judging sexual orientation from faces was better than chance. To test for effects of face spatial orientation (upright vs. upside-down), target gender, and participant sex, we performed a mixed-model analysis of variance (ANOVA) on accuracy with target gender as a repeated-measures factor and spatial orientation as a between-participants factor. Participant sex did not produce any significant main effects or interactions, consistent with Experiment 1 and previous work, e.g., [1], [3], and was dropped from analyses. As in Experiment 1, all analyses were repeated using d′ as the dependent measure; the results were unchanged. As in Experiment 1, hit rates (H_up_w_, H_up_m_, H_ud_w_, and H_ud_m_) and false alarm rates (FA_up_w_, FA_up_m_, FA_ud_w_, and FA_ud_m_) are reported in order to clarify the components of the accuracy scores we computed.

### Results and Discussion

**Figure 3:**
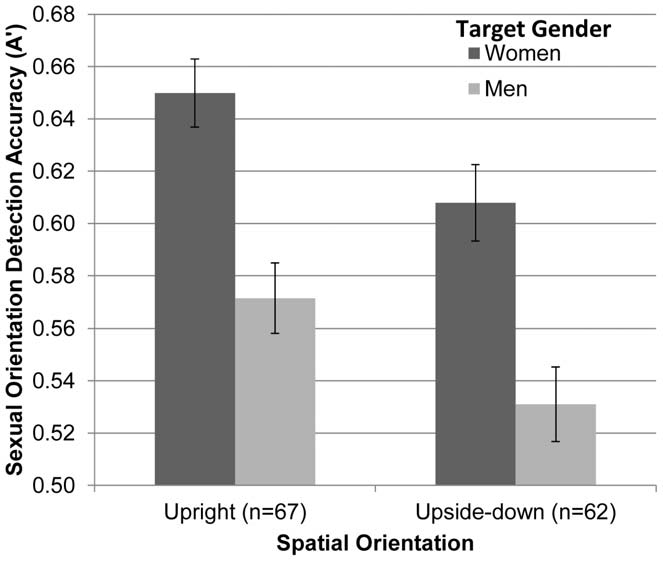
Accuracy of detecting sexual orientation from upright and upside-down faces (Experiment 2). Mean accuracy (A′) in judging sexual orientation from faces presented for 50 milliseconds as a function of the target’s gender and spatial orientation (upright or upside-down; Experiment 2). Judgments of upright faces are based on both configural and featural processing, whereas judgments of upside-down faces are based only on featural face processing. Error bars represent ±1 *SEM*.

As displayed in Figure 3, participants read sexual orientation significantly better than chance from upright faces of women (*Mean* A′_up_w_ = .65), *t*(66) = 11.49, *p*<.001, *d* = 1.40, and upright faces of men (*Mean* A′_up_m_ = .57), *t*(66) = 5.35, *p*<.001, *d* = .65.

Remarkably, participants read sexual orientation with above-chance accuracy from upside-down faces of women (*Mean* A′_ud_w_ = .61), *t*(61) = 7.38, *p*<.001, *d* = .94, and upside-down faces of men (*Mean* A′_ud_m_ = .53), *t*(61) = 2.17, *p*<.05, *d* = .28 (Figure 3). Because presenting faces upside-down severely disrupts configural face processing but has little [14], [15] or no detectable effect on featural face processing [8], [9], [10], [11], [12], [16], [17], [18], [19], [20], [21], [22], [23], [24], these results show that accurate judgments of women’s and men’s sexual orientation were possible even when face processing was largely restricted to featural face information.

Does configural face processing boost accuracy of sexual orientation judgments above the accuracy observed when judgments are primarily limited to featural face processing (i.e., during the upside-down trials)? Yes. The ANOVA yielded a main effect of spatial orientation, *F*(1, 127) = 7.67, *p* = .006, *d* = 0.49, indicating that for both faces of women and men, participants were significantly more accurate at reading sexual orientation from upright faces than from upside-down faces. The increase in accuracy for judging faces presented upright suggests that the ability to read sexual orientation from women’s and men’s faces *does* significantly rely on configural face processing, in addition to featural face processing.

Replicating Experiment 1, the ANOVA yielded a main effect of target gender, *F*(1, 127) = 37.71, *p*<.001, *d* = 0.77, indicating that women’s faces, regardless of spatial orientation, were judged more accurately than men’s faces. As in Experiment 1, hit rates did not significantly differ for judgments of women’s and men’s faces, but false alarm rates were significantly lower for judgments of women’s faces than for men’s faces (see Table 1 for hit and false alarm rates as well as inferential statistics). There was no evidence to suggest an interaction between spatial orientation and target gender, *F*(1, 127) = 0.003, *ns*.

**Table 1:** Hit and False Alarm Rates for Snap Judgments of Sexual Orientation in Experiment 2.

		Face Gender		
		Women	Men	
Spatial Orientation	Accuracy Component	*Mean* (*SD*)	*Mean* (*SD*)	Paired-*t*
Upright	Hit Rate	.36 (0.13)	.37 (0.13)	−0.45
	False Alarm Rate	.22 (0.15)	.30 (0.16)	−4.52**
Upside-down	Hit Rate	.38 (0.17)	.40 (0.15)	−1.78†
	False Alarm Rate	.27 (0.18)	.38 (0.17)	−6.05**

Table 1 Note: Upright *n* = 67; upside-down *n* = 62. Hit rates are the proportion of gay faces correctly identified as gay; false alarm rates are the proportion of straight faces incorrectly identified as gay.

† *p*<.10.

** *p*<.001.

## Discussion

The present research was the first attempt to determine the roles that featural and configural face processing play in snap judgments of sexual orientation from faces. Participants were able to judge the sexual orientation of women’s and men’s faces with above-chance accuracy, but their ability to do so was significantly impaired when the photographs were presented upside-down. These results elucidate the processes by which sexual orientation is judged from the face in several ways. First, because accuracy of sexual orientation judgments was appreciably reduced for upside-down (vs. upright) faces of both women and men – a situation in which configural face processing is strongly inhibited [8], [9], [10], [11], [12], [14], [15], [16], [17], [18], [19], [20], [21], [22], [23], [24] – these data show that configural face processing contributes to judgment accuracy. Accordingly, as experiments aim to examine the precise face characteristics that differentiate gay and straight faces, researchers should look for differences in relationships among facial features as well as differences in features themselves.

Could the decrease in judgment accuracy for upside-down faces reflect a decrease in featural face processing? It seems unlikely. Meta-analyses or review papers repeatedly find robust effects of facial inversion on configural face processing, e.g., [9], [11], [15], [21], [24], but any effects of facial inversion on featural face processing are small and rare [14], [15]. Moreover, researchers who did find facial inversion effects using faces manipulated in featural content attributed the effects to their observation that the specific featural differences they created “also affect [configural] relations with the rest of the face” [15] (p. 50). That is, these researchers reasoned that the effects of facial inversion that they observed for faces ostensibly differing in figural information were actually caused by unintentional configural differences caused by feature changes (such as a circular eye replacing an elliptical eye and therefore changing the eye-nose distance). Accordingly, in our view, as in previous research, e.g., [12], [21], [24], it seems likely that the effects of facial inversion are mostly, if not entirely, attributable to decrements in configural face processing and not to decrements in featural face processing.

Moreover, the finding that judgment accuracy remained above chance for upside-down faces strongly suggests that sexual orientation can be inferred from featural processing alone. Evidence suggests that if a trait can be inferred from featural processing alone, it may be inferred spontaneously and unintentionally in everyday life [12], [16], [17]; see also [41]. Thus, the present results imply that in casual interactions, people may unwittingly accurately perceive others’ sexual orientation from brief glances at their faces (see [3], [41], [42], [43]). If so, it would appear that minority sexual orientation is not the concealed stigma that many argue it is. Indeed, the need to protect gay people from discrimination would seem increasingly urgent to the extent that minority sexual orientation is tacitly inferred from aspects of personal appearance that are routinely available for inspection (e.g., faces). Although the present experiments deal primarily with whether above-chance accuracy in snap judgments of sexual orientation from faces *can* occur and *how* faces are processed to give rise to such judgments, it does so in an experimental setting wherein individuals are instructed to make forced-choice judgments of sexual orientation. Recent work, e.g., [41], [42] suggests that inferences of sexual orientation need not depend on the explicit instructions to judge faces as gay or straight. Nonetheless, a relatively unexplored question that is ripe for future research involves the external validity of these effects – do snap judgments of sexual orientation from faces occur in real-life settings? Additionally, what are the downstream consequences of snap judgments of sexual orientation, for example, on the perceiver’s feelings, thoughts, and behaviors towards the target?

Do sexual orientation judgments rely on category or identity person information? Previous work has postulated that configural face processing is necessary for judging identity person information (e.g., famous vs. not famous) but that only featural face processing is necessary for judging category person information (e.g., male vs. female) [12], [14]. Here, we found that configural face processing improved accuracy of sexual orientation judgments, but was not necessary to enable above-chance judgment accuracy. Given that sexual orientation is a less obvious category, compared to race or sex, it may prompt the use of both category (featural) and individuating (configural) face information. This is in contrast to processing of faces representing more physiognomically obvious categories (e.g., sex) that may be clearly ascertained by featural cues alone. Future research should examine whether all faces invoke, and all perceivers rely on, both featural and configural processing for sexual orientation judgments, or whether only some faces invoke and/or only some perceivers rely on configural face processing in addition to featural face processing.

Second, the results indicate that the process of reading sexual orientation from faces of women is notably easier than the process of reading sexual orientation from faces of men. That is, participants read sexual orientation more accurately from women’s faces than from men’s faces (*Mean* difference in A′ = .078, or approximately 7.8 percentage points). Though this difference was suggested by casual comparisons of results across papers (i.e., [1], [2] vs. [3]), the present experiment was the first in which participants judged faces of both genders, and thus the first experiment in which a direct comparison of accuracy for women’s and men’s faces could be computed. Moreover, this difference persisted regardless of spatial orientation, suggesting that women’s sexual orientation is more obvious than men’s both in individual facial features and in facial configuration. The prospect of distinct processes for extracting sexual orientation from women’s and men’s faces is intriguing, yet not entirely surprising. The face is assumed to reflect experiences. Men and women differ in their subjective experiences and overt expressions of romantic love and sexual desire, as well as their biological (neurophysiological and hormonal) underpinnings, e.g., [25], [26], [27], [28], [29], [30], [31]. The current findings suggest that facial expressions of sexual orientation also differ by gender.

The present research is the first to demonstrate (a) that configural face processing significantly contributes to perception of sexual orientation, and (b) that sexual orientation is inferred more easily from women’s vs. men’s faces. In light of these findings, it is interesting to note the popular desire to learn to read faces like books, e.g., [44]. Considering how challenging it is to read a book upside-down, it seems that we read faces *better* than we read books.
